# Analysis of L-arginine:glycine amidinotransferase-, creatine- and homoarginine-dependent gene regulation in the murine heart

**DOI:** 10.1038/s41598-020-61638-3

**Published:** 2020-03-16

**Authors:** Märit Jensen, Christian Müller, Chi-un Choe, Edzard Schwedhelm, Tanja Zeller

**Affiliations:** 1 University Heart and Vascular Centre Hamburg, Clinic for Cardiology, University Medical Centre Hamburg-Eppendorf, Hamburg, 20246 Germany; 2 German Centre for Cardiovascular Research (DZHK e.V.), partner site Hamburg/Kiel/Lübeck, Hamburg, 20246 Germany; 3 Department of Neurology, University Medical Centre Hamburg-Eppendorf, Hamburg, 20246 Germany; 4 Institute of Clinical Pharmacology and Toxicology, University Medical Centre Hamburg-Eppendorf, Hamburg, 20246 Germany

**Keywords:** Transcriptomics, Gene expression, Cardiovascular genetics, Cardiovascular diseases

## Abstract

L-arginine:glycine amidinotransferase (AGAT) and its metabolites creatine and homoarginine (HA) have been linked to cardiovascular pathologies in both human and murine studies, but the underlying molecular mechanisms are poorly understood. Here, we report the first analysis of heart transcriptome variation using microarrays in an AGAT-deficient (AGAT^−/−^) mouse model to evaluate AGAT-, creatine- and HA-dependent gene regulation. Our data revealed significant differences of gene expression between AGAT^−/−^ and wild-type (WT) mice, affecting cardiac energy metabolism (*Fbp2*, *Ucp2*), cardiac hypertrophy and fibrosis (*Nppa*, *Ctgf*), immune response (*Fgl2*), and the conduction system of the heart (*Dsc2*, *Ehd4*, *Hcn2, Hcn4*, *Scn4a, Scn4b*). All of these genes being expressed on WT level in creatine-supplemented mice. Using *in silico* analysis based on the GEO database we found that most of these candidate genes (*Ctgf*, *Dsc2*, *Fbp2*, *Fgl2*, *Hcn2*, *Nppa*)  revealed significant alterations in a WT mouse model of myocardial infarction underlining a pathophysiological relationship between AGAT metabolism and cardiovascular disease.

## Introduction

Cardiovascular disease (CVD) is one of the major causes of death and hospitalization in the world^1^. Current risk stratification is mainly based on classical risk factors such as hypertension, smoking, hypercholesterolemia, obesity and diabetes. However, these factors do not include the molecular and metabolic factors which reflect the complex and heterogenic etiology of CVD. Several epidemiological studies in the cardiovascular field identified novel biomarkers to predict clinical outcome and provide a better understanding of disease progression. In particular, metabolic pathways in CVD represent potential new valuable targets for drug therapy^2,3^.

The L-arginine derivative L-homoarginine (HA) and the nitrogenous organic acid creatine are part of one such pathway and have been investigated with regard to CVD. HA was identified as a prognostic factor for CVD. Several studies revealed a correlation between low HA plasma levels and an increased cardiovascular mortality, e.g., after myocardial infarction (MI), in heart failure and after ischemic stroke^4–6^. Moreover, HA is involved in nitric oxide (NO) metabolism and inhibits alkaline phosphatase at high concentrations linking it to vascular function and atherosclerosis^7,8^. Creatine on the other hand, plays a key role in cardiac energy metabolism and acts as a rapidly available energy buffer by its involvement in the recycling of adenosine triphosphate (ATP)^9^. It has been shown that all major components of this system including creatine are down-regulated in the failing heart^10,11^.

The common enzyme for endogenous HA and creatine formation is the L-arginine:glycine amidinotransferase (AGAT; EC: 2.1.4.1). In humans, single-nucleotide polymorphisms (SNPs) within the *AGAT* gene are associated with variations of HA plasma concentrations^12,13^. For the leading SNP rs1288775 (AGAT missense), homozygous allele carriers of the minor allele had significantly higher plasma HA compared with heterozygous or homozygous allele carriers of the major allele, representing a gene dose-dependent effect^12,13^. Previously we have generated AGAT-deficient (AGAT^−/−^) mice which showed whole body HA and creatine deficiency^12,14^. These AGAT^−/−^ mice exhibited a cardiovascular phenotype of low left ventricular end-systolic pressure (LVESP), impaired contractility and relaxation, as well as a lower maximal heart rate and contractile reserve in response to dobutamine infusion compared to wild-type (WT) mice^15^. Creatine supplementation corrected LVESP, while HA supplementation rescued all other hemodynamic parameters^15^. In a model of post-myocardial infarction, HA supplementation normalized several cardiac parameters in WT mice (i.e., relaxation, cardiac reserve)^16^. However, data on the underlying molecular mechanisms and signal transduction pathways in the AGAT metabolism are scant.

In this study we analysed the cardiac transcriptome of left ventricular tissue of WT mice, untreated AGAT^−/−^ mice and AGAT^−/−^ mice with creatine (AGAT^−/−^Cr) or HA (AGAT^−/−^HA) supplementation. The aim of our study was to identify molecular pathways and cardiovascular candidate genes related to AGAT and creatine or HA supplementation. Additionally, candidate genes were evaluated in a WT mouse model of MI using GEO data.

## Results

### Cardiac gene expression is altered in AGAT^−/−^ mice

**Figure 1:**
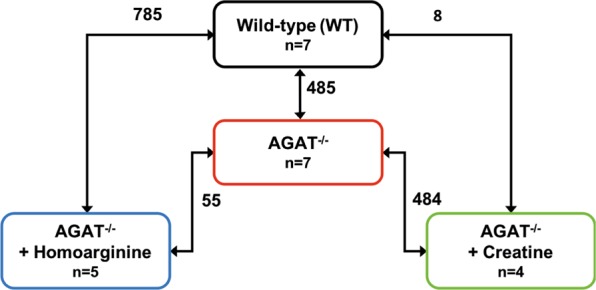
Number of differentially expressed genes between the groups in murine heart tissue. Transcriptome profiling was performed using the Affymetrix Mouse GeneChip 1.0 ST Array. Each line indicates the comparison of the respective two groups and the number of significantly regulated genes. Significance level: False-Discovery-Rate (FDR) ≤ 0.05. Abbreviations: WT, wild-type; AGAT^−/−^, AGAT knock-out; n, number of animals.

We performed a transcriptome analysis of left ventricular tissue of WT, AGAT^−/−^, AGAT^−/−^Cr and AGAT^−/−^HA mice in heart samples. The number of differentially expressed genes was evaluated for each comparison (Fig. 1).

**Figure 2:**
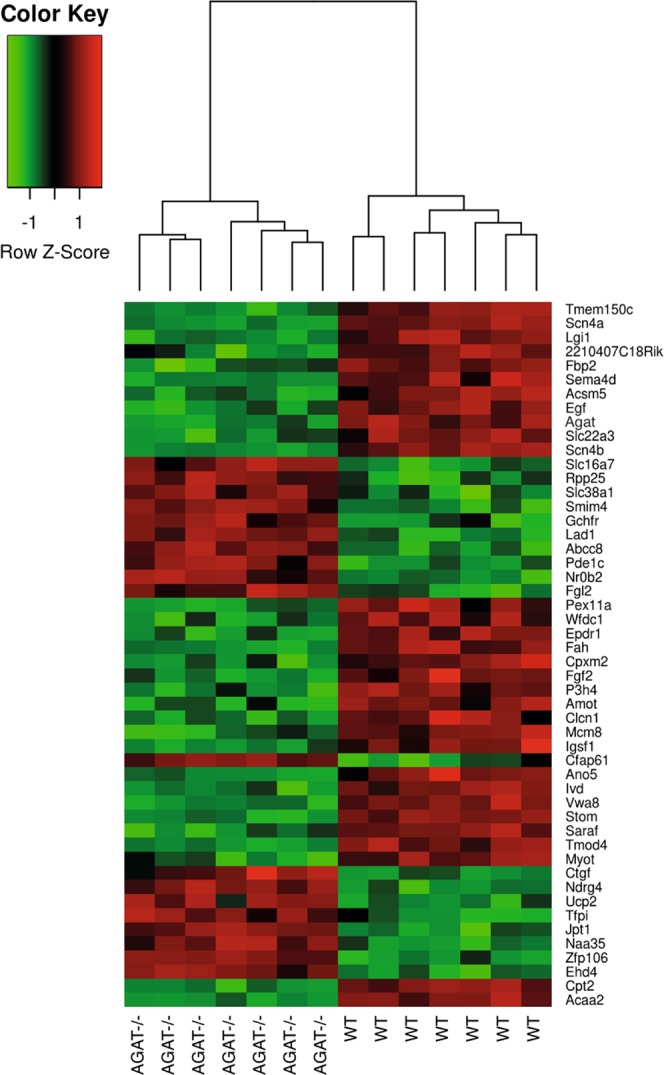
Heatmap of gene expression values depicting clustering of genes between WT and AGAT^−/−^ heart tissue samples. The heatmap is based on the expression of mRNAs for the set of the top 50 significant genes. Low to high expression is represented by a change of color from green to red, respectively. Abbreviations: AGAT^−/−^, AGAT knock-out mice; WT, wild-type mice.

Comparison of WT and AGAT^−/−^ mice revealed 485 significantly de-regulated genes (FDR ≤ 0.05; see Supplementary Table S1). The top 20 most up- and down-regulated genes are shown in Table 1. A heatmap of the top 50 de-regulated genes is displayed in Fig. 2. Differentially expressed genes were enriched for pathways (WikiPathways) involved in energy metabolism such as mitochondrial LC-fatty acid beta-oxidation (P = 1.76 × 10^−9^), fatty acid beta-oxidation (P = 4.12 × 10^−9^) and glycogen metabolism (P = 8.03 × 10^−6^). At the level of individual genes, the majority of genes involved in beta-oxidation were down-regulated in AGAT^−/−^ mice. An enrichment of genes involved in cardiac calcium regulation (P = 4.32 × 10^−7^) indicates a relation of these genes to cardiovascular metabolism (see Supplementary Fig. S1).

**Table 1:** Top 20 differentially expressed genes between WT and AGAT^−/−^ mice in heart tissue. False-Discovery-Rate ≤ 0.05. Abbreviations: FC, fold change.

Gene	Gene name	P Value	FC
*Scn4a*	sodium channel, voltage-gated, type IV, alpha	3.02 × 10^−13^	−3.22
*Scn4b*	sodium channel, type IV, beta	1.47 × 10^−12^	−3.74
*Tmod4*	tropomodulin 4	8.93 × 10^−11^	−1.76
*Tmem150c*	transmembrane protein 150 C	9.97 × 10^−11^	−2.96
*Fah*	fumarylacetoacetate hydrolase	1.08 × 10^−10^	−2.36
*Agat*	L-arginine:glycine amidinotransferase	1.18 × 10^−10^	−2.19
*Lgi1*	leucine-rich repeat LGI family, member 1	2.07 × 10^−10^	−3.10
*Lad1*	ladinin	2.63 × 10^−10^	2.17
*Stom*	stomatin	1.12 × 10^−9^	−1.41
*Zfp106*	zinc finger protein 106	1.31 × 10^−9^	1.49
*Egf*	epidermal growth factor	6.62 × 10^−9^	−1.55
*Ndrg4*	N-myc downstream regulated gene 4	9.12 × 10^−9^	1.44
*Vwa8*	von Willebrand factor A domain containing 8	1.20 × 10^−8^	−1.35
*Ano5*	anoctamin 5	1.78 × 10^−8^	−2.27
*Slc16a7*	solute carrier family 16 (monocarboxylic acid transporters), member 7	1.97 × 10^−8^	1.70
*Ivd*	isovaleryl coenzyme A dehydrogenase	2.54 × 10^−8^	−1.41
*Hn1*	hematological and neurological expressed sequence 1	2.60 × 10^−8^	1.41
*Nr0b2*	nuclear receptor subfamily 0, group B, member 2	3.93 × 10^−8^	1.47
*Slc22a3*	solute carrier family 22 (organic cation transporter), member 3	3.99 × 10^−8^	−1.66
*Acsm5*	acyl-CoA synthetase medium-chain family member 5	4.32 × 10^−8^	−1.88

Creatine and HA have beneficial effects in AGAT^−/−^ mice on cardiac function^15^. Therefore, we analysed gene regulation in hearts of AGAT^−/−^ mice after either creatine or HA supplementation. We identified eight genes that were significantly different between WT and AGAT^−/−^Cr littermates (i.e., 2% of AGAT-dependent de-regulated genes; FDR ≤ 0.05). Thus, it can be assumed that the majority of de-regulated genes in the heart are creatine dependent. In AGAT^−/−^HA mice, 785 (FDR ≤ 0.05) genes were significantly de-regulated compared with expression levels in WT mice. 281 AGAT-dependent genes (i.e., 58%) remained differentially expressed in AGAT^−/−^HA mice compared to WT mice. Considering that more genes were differentially expressed between WT and AGAT^−/−^HA mice than between WT and AGAT^−/−^ littermates (785 vs. 485 genes, respectively), it can be assumed that HA supplementation and AGAT^−/−^ have an additive effect on changes seen for gene expression as compared with WT animals.

### Network analysis reveals AGAT-dependent candidate genes

**Figure 3:**
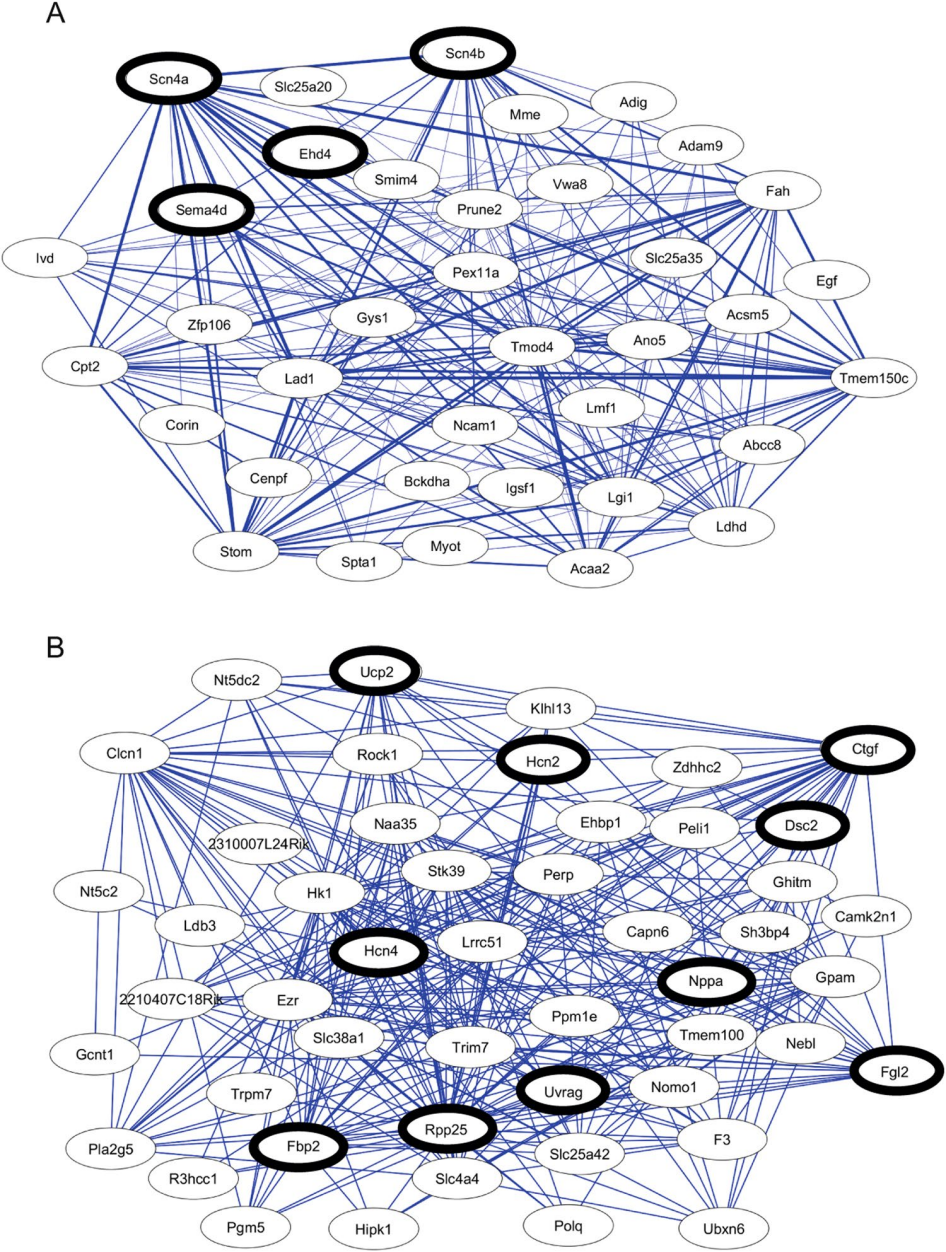
Network analysis between WT and AGAT^−/−^ mice in heart tissue. Differentially expressed genes were clustered into modules of highly co-expressed genes. The networks with the strongest associations are shown (A, P = 1.07 × 10^−8^; B, P = 4.39 × 10^−8^). Candidate genes (black circles) were selected based on 1.) known association with CVD and 2.) statistical significance.

To detect important genes within the AGAT metabolism, we constructed modules of highly correlated genes by weighted correlation network analysis (WGCNA). Figure 3 shows the top two networks with the strongest association to the AGAT knock-out. The entire list of genes in Network 1 and 2 can be found as Supplementary Tables S2 and S3. Of these genes, candidates were selected based on 1.) known association with CVD and 2.) statistical significance (Table 2).

**Table 2:** AGAT-related candidate genes. Genes were selected based on 1. known association with CVD^+^ and 2. statistical significance^#^. For genes selected based on known association with CVD the according reference is given. False-Discovery-Rate ≤ 0.05. Abbreviations: FC, fold change; NS, not significant.

Gene	WT vs. AGAT^−/−^		WT vs. AGAT^−/−^Cr		WT vs. AGAT^−/−^HA		References
	P Value	FC	P Value	FC	P Value	FC	
*Ctgf*^+^	5.21 × 10^−7^	1.78	3.1 × 10^−3^	−1.33	1.36 × 10^−7^	1.83	Ohnishi *et al*.^23^, Ahmed *et al*.^24^, Oemar *et al*.^25^, Koshman *et al*.^26^
*Dsc2*^+^	4.33 × 10^−5^	1.35	NS	−1.07	NS	1.10	Sun *et al*.^35^, Brodehl *et al*.^36^
*Ehd4*^+^	6.03 × 10^−8^	1.36	NS	−1.04	3.15 × 10^−6^	1.34	Dun *et al*.^34^
*Fbp2*^*#*^	1.06 × 10^−7^	−1.57	1.64 × 10^−2^	−1.12	4.66 × 10^−7^	−1.46	—
*Fgl2*^+^	3.1 × 10^−7^	1.44	2.69 × 10^−3^	−1.2	3.65 × 10^−2^	1.15	Zhenzhong *et al*.^37^
*Hcn2*^+^	1.48 × 10^−5^	1.33	9.67 × 10^−3^	−1.17	2.57 × 10^−3^	1.2	Robinson *et al*.^30^, Vaccari *et al*.^32^
*Hcn4*^+^	2.93 × 10^−4^	−1.42	NS	−1.07	8.8 × 10^−3^	−1.28	Robinson *et al*.^30^, DiFrancesco *et al*.^31^, Baruscotti *et al*.^33^
*Nppa*^+^	1.17 × 10^−5^	1.93	NS	1.02	1.16 × 10^−2^	1.55	Wang *et al*.^22^
*Rpp25*^*#*^	8.71 × 10^−8^	1.69	NS	1	2 × 10^−5^	1.57	—
*Scn4a*^+*,#*^	3.02 × 10^−13^	−3.22	NS	−1.13	6.63 × 10^−11^	−2.81	Coronel *et al*.^27^, Lau *et al*.^28^
*Scn4b*^+*,#*^	1.47 × 10^−12^	−3.74	NS	1.12	6.13 × 10^−8^	−3.34	Li *et al*.^29^
*Sema4d*^+^	8.91 × 10^−8^	−1.36	2.76 × 10^−2^	−1.13	2.4 × 10^−7^	−1.43	Willner *et al*.^44^
*Ucp2*^*+*^	3.49 × 10^−7^	1.85	NS	−1.13	8.67 × 10^−6^	1.53	Saleh *et al*.^20^, Akhmedov *et al*.^21^
*Uvrag*^*+*^	3.45 × 10^−4^	1.14	NS	−1.03	NS	1.05	Song *et al*.^45^

We first looked for genes with association to the cardiovascular system that have been discussed in literature. Among those genes are six well known CVD-associated genes. These include sodium voltage-gated channel alpha subunit 4 (*Scn4a*, P = 3.02 × 10^−13^), sodium voltage-gated channel beta subunit 4 (*Scn4b*, P = 1.47 × 10^−12^), and hyperpolarization-activated cyclic nucleotide-gated ion channel 4 (*Hcn4*, P = 2.93 × 10^−4^), which were all down-regulated in AGAT^−/−^ mice. Connective tissue growth factor (*Ctgf*, P = 5.21 × 10^−7^), natriuretic peptide type A (*Nppa*, P = 1.17 × 10^−5^) and hyperpolarization-activated cyclic nucleotide-gated ion channel 2 (*Hcn2*, P = 1.48 × 10^−5^) were found to be up-regulated in AGAT^−/−^ mice.

In addition, the results revealed that uncoupling protein 2 (*Ucp2*, P = 3.49 × 10^−7^), EH domain-containing protein 4 (*Ehd4*, P = 6.03 × 10^−8^), Semaphorin-4D (*Sema4d*, P = 8.91 × 10^−8^), fibrinogen like 2 *(Fgl2*, P = 3.1 × 10^−7^*)*, Desmocollin 2 (*Dsc2*, P = 4.33 × 10^−5^) and UV radiation resistance associated gene *(Uvrag*, P = 3.45 × 10^−4^) were already linked to cardiovascular pathologies (see Table 2).

The top two significant genes within network 1 and 2 were *Scn4a* and *Scn4b* as well as ribonuclease P protein subunit p25 (*Rpp25*, P = 8.71 × 10^−8^) and fructose-1,6-bisphosphatase isozyme 2 (*Fbp2*, P = 1.06 × 10^−7^).

**Figure 4:**
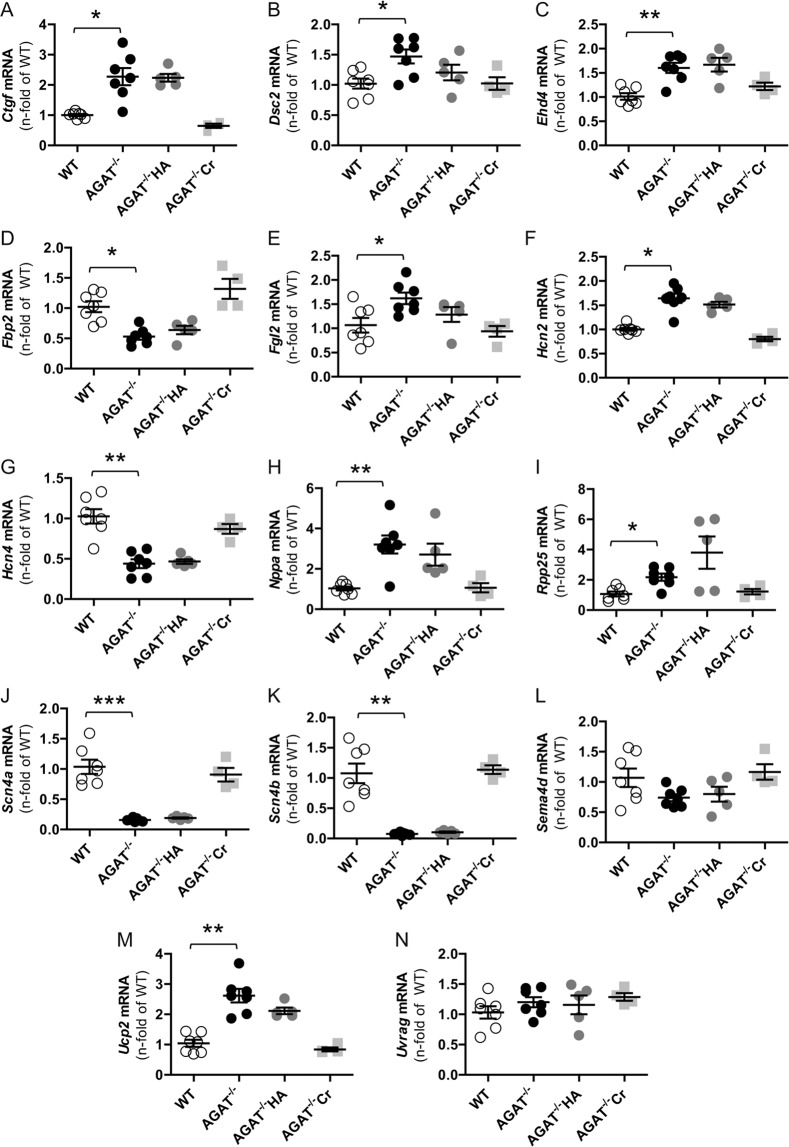
Validation of candidate genes by qPCR. Relative mRNA expression of candidate genes in the heart identified based on known association to the cardiovascular system and statistical significance. Each data point represents an individual mouse. Values are expressed as mean ± SEM. *P < 0.05; **P < 0.01 and ***P < 0.001 versus WT, Kruskal-Wallis-Test. WT (n = 7), AGAT^−/−^ (n = 7), AGAT^−/−^HA (n = 5), AGAT^−/−^Cr (n = 4).

The majority of our candidate genes have been validated by qPCR. No significant de-regulation was found for *Sema4d* and *Uvrag* (Fig. 4). Thus, we excluded these genes from further analysis. As expected from microarray analysis, comparison of WT and AGAT^−/−^Cr mice revealed that creatine supplementation leads to a restoration of gene expression levels towards WT levels. Comparing the expression levels of candidates between WT and AGAT^−/−^HA mice, we found that most candidate genes remained differentially expressed.

### Candidate genes are de-regulated in a mouse model of myocardial infarction

**Figure 5:**
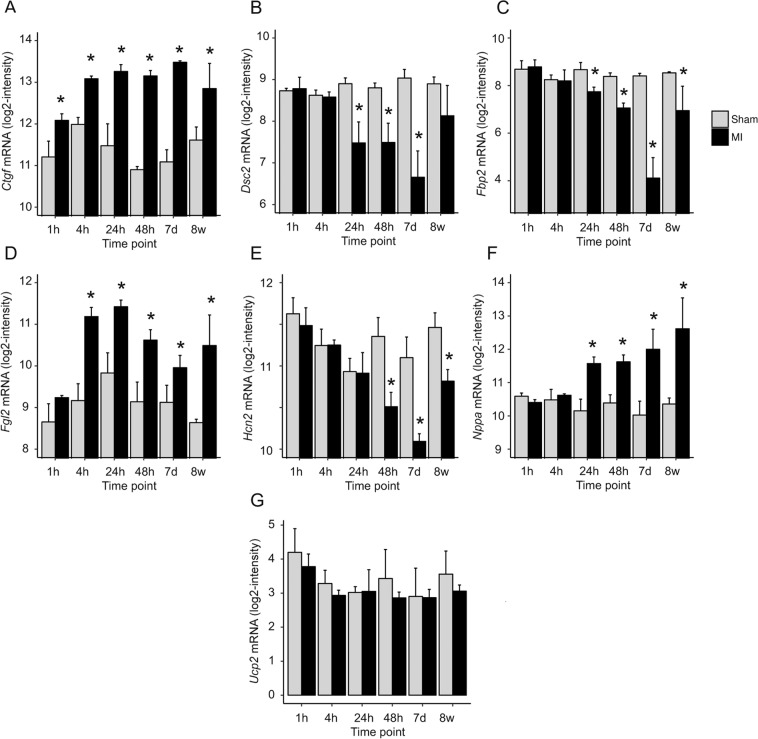
*In silico* gene expression analysis of candidate genes after MI. mRNA expression of *Ctfg*, *Dsc2*, *Fbp2*, *Fgl2*, *Hcn2, Nppa* and *Ucp2* was measured in log2 microarray light intensity at 1 h, 4 h, 24 h, 48 h, 7 days and 8 weeks after experimentally induced MI. The GEO dataset GSE775 (Mouse model of myocardial infarction) was used. *P < 0.05 versus sham. Abbreviations: MI, myocardial infarction.

Since AGAT and HA/creatine metabolism has been linked to MI and heart failure in both human and mice^4–6,16^, we performed *in silico* gene expression analysis in a mouse model of MI to test our candidate genes in response to MI. For this, we used the publicly available GEO dataset GSE775^17^. WT mice were subjected either to MI by ligation of the left coronary artery or the sham operation, and gene expression between both groups was compared at six time points after MI. Among the 12 validated candidate genes, expression data were available for *Ctgf*, *Dsc2*, *Fbp2*, *Fgl2*, *Hcn2*, *Nppa* and *Ucp2* (Fig. 5). *Ctgf*, *Fgl2* and *Nppa* mRNA expression was significantly up-regulated at various time points post-MI, while gene expression of *Dsc2*, *Fbp2* and *Hcn2* was down-regulated in MI mice compared with sham-operated mice. Expression levels of *Ucp2* were not altered in the infarcted heart.

## Discussion

The identification of unknown molecular mechanisms in CVD is needed to develop novel therapeutic strategies. In this context, the enzyme AGAT is of great importance since it is responsible for endogenous creatine and HA formation^5^. A lack of creatine results in altered cardiac energy metabolism^10,11^ and low HA plasma levels are associated with poor prognosis in CVD^18^. In this work we present transcriptomic variations linking AGAT, creatine and HA with its implications in cardiac (dys)function and CVD. As a main result we found that AGAT^−/−^ mice exhibited altered gene expression related to energy metabolism (*Fbp2*, *Ucp2*), cardiac hypertrophy and fibrosis (*Nppa*, *Ctgf*), immune response (*Fgl2*), and the conduction system of the heart (*Dsc2*, *Ehd4*, *Hcn2, Hcn4*, *Scn4a, Scn4b*). Notably, all of these genes being expressed on WT level in creatine-supplemented mice.

Our data revealed a widespread effect of the AGAT^−/−^ genotype on cardiac gene expression. This data complements with our previous cardiac phenotype studies that showed an impaired cardiac function in AGAT^−/−^ mice^15^. Pathway analysis of genes differentially expressed between WT and AGAT^−/−^ animals revealed that genes were enriched in energy metabolism pathways, such as mitochondrial LC-fatty acid beta-oxidation, fatty acid beta-oxidation and glycogen metabolism. This link between AGAT deficiency and energy metabolism, evidenced by diminished intracellular energy stores i.e., ATP and phosphocreatine (PCr) in brain and skeletal muscle, had been described previously^9,14^. However, while PCr was completely absent in AGAT^−/−^ hearts, ATP remained at WT level^15^. Thus, AGAT^−/−^ mice seem to be able to maintain cardiac ATP production and storage through ATP synthesis pathways independent of the creatine/PCr system. Our analysis did not uncover alternative mechanisms of ATP synthesis as the majority of genes involved in beta-oxidation were down-regulated in AGAT^−/−^ mice. Therefore, further research is required to assess which mechanisms of ATP synthesis are responsible.

In addition, pathway analysis suggested an explanation for the observed association of AGAT deficiency and cardiac contractility, as cardiac calcium regulation was represented within the top ten pathways. Calcium is a critical regulator of cardiac myocyte function and the essential link in excitation contraction coupling^19^. De-regulation of cytoplasmatic calcium leads to cardiac dysfunction, i.e., impaired contractility and relaxation and reduced inotropic reserve. This finding needs to be further experimentally addressed. Nevertheless, we provided a first hint to molecular mechanisms that could explain altered cardiac function in our AGAT^−/−^ mice as shown previously^15^.

Inspecting all genes included in the network analysis of differentially expressed genes between WT and AGAT^−/−^ mice, we found 14 candidate genes for changes in cardiovascular function of AGAT^−/−^ animals (*Ctgf*, *Dsc2*, *Ehd4*, *Fbp2*, *Fgl2, Hcn2*, *Hcn4*, *Nppa*, *Rpp25*, *Scn4a*, *Scn4b*, *Sema4d*, *Ucp2* and *Uvrag)*. We excluded *Sema4d* and *Uvrag* from further analysis as validation by qPCR showed no significant AGAT-dependent regulation of these genes.

AGAT itself is an important regulator of energy homeostasis. Other individual energy related genes that might be additionally affected by AGAT deficiency comprise *Fbp2*, encoding for a gluconeogenesis enzyme, and *Ucp2*, encoding for a member of the family of inner mitochondrial membrane proteins. UCP2 is involved in lipid metabolism and plays a role in the control of mitochondria-derived reactive oxygen species (ROS)^20^. Increasing evidence suggests that UCPs could play a protective role in myocardial function by reducing mitochondrial ROS generation and cardiomyocyte apoptosis^21^. Up-regulation of *Ucp2* mRNA might be an adaptive mechanism, allowing the cardiac myocytes to compensate for the higher ROS production in AGAT^−/−^ mice.

We showed that two genes (*Nppa*, *Ctgf*) involved in cardiac hypertrophy and fibrosis were de-regulated in AGAT^−/−^ animals. The protein encoded by the *Nppa* gene is atrial natriuretic peptide (ANP), which in turn predicts cardiovascular outcomes and is a biomarker of hypertrophy^22^. An up-regulation of *Nppa* mRNA may indicate the presence of a molecular programme of hypertrophy. However, this finding has to be critically evaluated since another study showed that not all molecular markers of cardiac hypertrophy were consistently elevated in AGAT^−/−^ mice, i.e., unchanged mRNA expression of brain natriuretic peptide (BNP) and beta myosin heavy chain (β-MHC) and reduced mRNA expression of alpha skeletal actin (α-SA)^15^. Our results additionally identified *Ctgf* mRNA to be significantly up-regulated in response to the knock-out. Previous studies showed that the encoded protein CTGF is up-regulated in cardiac cells after MI and in advanced atherosclerosis^23–25^. In addition, CTGF may be a key regulator of fibrosis during remodelling and progression to heart failure^26^. It does demonstrate that AGAT deficiency may be associated with fibrosis.

Another large proportion of candidate genes de-regulated in AGAT deficiency are associated with the cardiac conduction system. Pacemaker activity and electrical propagation may be directly affected by ion channel de-regulation (*Hcn2*, *Hcn4*, *Scn4a*, *Scn4b*) or indirectly affected (*Ehd4*) or may be affected by structural alterations (*Dsc2*). *Scn4a* and *Scn4b* mutations and expression changes have been shown to be associated with arrhythmia and tightly regulated expression of these channels is required for physiological conduction^27–29^. Both channels were significantly down-regulated in AGAT^−/−^ mice. *Hcn2* and *Hcn4* are coding for HCN channels mediating the so-called funny current (I_f_) which plays a key role in the generation and modulation of cardiac automaticity^30^. HCN4 is highly expressed in the sinoatrial node and responsible for heart rhythm generation through spontaneous diastolic depolarization^31^. Though HCN2 is not as robustly expressed in the heart, it additionally contributes to spontaneous rhythmic activity^32^. The cardiac-specific knock-out of *Hcn4* in mice leads to the development of bradycardia as pacemaking activity in these animals solely relies on calcium clock mechanisms^33^. In line with those data, AGAT^−/−^ mice in which *Hcn4* mRNA is down-regulated exhibited a lower maximal heart rate compared to WT littermates^15^. *Ehd4* mRNA was up-regulated in AGAT^−/−^ mice. Given that previous studies revealed that the EHD4 protein is enhanced in cells from the infarcted heart and related to reduced expression of cardiac sodium channels causing cardiac arrythmias^34^, the described up-regulation may indicate a susceptibility of AGAT^−/−^ mice to arrythmias. Structural alterations in the heart may also cause cardiac arrythmias. Mutations in the *Dsc2* gene, encoding a desmosome-related protein, lead to abnormalities in cardiac structure and are involved in the development of arrhythmogenic cardiomyopathy^35,36^. *Dsc2* mRNA was significantly de-regulated in AGAT^−/−^ littermates.

We further observed de-regulated genes in AGAT^−/−^ mice that are not commonly associated with CVD. However, there is evidence that these genes may influence cardiac function. In a diabetes mouse model *Fgl2* gene silencing inhibits apoptosis and improves heart function by probably modulating immune response^37^. *Rpp25* that was selected based on statistical significance needs further validation of an involvement in CVD.

To confirm previously described associations of our candidates with CVD, we performed *in silico* analysis in an experimental mouse model of MI. Using the available data, we were able to show a de-regulation of *Ctgf*, *Dsc2*, *Fbp2*, *Fgl2*, *Hcn2* and *Nppa* at various time points after MI. These results are compatible with the idea that a de-regulation of our candidate genes plays a role in cardiac (dys)function and CVD.

Considering that both creatine and HA are involved in cardiac (dys)function, we performed additional analyses to differentiate between creatine- and HA-dependent effects on the gene expression. We found that on gene expression level creatine rather than HA restored cardiac gene expression towards the WT. Previously, our group showed that AGAT deficiency results in disturbed energy metabolism in the muscle^9,38^. As expected from these studies, a de-regulation of energy-related genes in the heart was reversible by creatine supplementation. Regarding cardiac function, our AGAT^−/−^ mice exhibited a cardiovascular phenotype of LVESP, impaired contractility and relaxation, as well as a lower maximal heart rate and contractile reserve^15^. LVESP was corrected by creatine supplementation which may be associated with a creatine-dependent regulation of *Ctgf* and *Nppa*. This finding is important as LVESP also affects the peripheral cardiovascular system.

However, all other measured parameters of cardiac function in AGAT^−/−^ mice were normalized by HA supplementation. Additionally, HA supplementation in a WT mouse model of post-MI heart failure attenuated the impaired cardiac function underlining a positive effect of HA on the cardiovascular system^16^. On gene expression level, our data cannot explain the underlying mechanisms of HA effect on cardiac function. We therefore assume that HA may influence the cardiac system through other mechanisms such as post-translational changes or may mainly affect other areas of the heart except the left ventricle as described below.

A limitation of our study was the exclusive use of left ventricular heart tissue. It can thus be assumed that some of the transcriptomic changes that determine the cardiac phenotype of AGAT^−/−^ mice in the other chambers have been systematically overlooked. In addition, the correlation of gene expression and protein abundance is a critical point in research, especially when using high-throughput methods. Therefore, further analysis and detailed assessment of target genes identified in our transcriptome analyses such as protein and/or metabolome analysis have to be performed. Moreover, experiments in AGAT^−/−^ CVD models (e.g., MI or heart failure) will directly link AGAT deficiency with CVD.

Taken together, our study provides first transcriptomic data on the molecular background of the AGAT metabolism and the influence of creatine and HA on molecular pathways regarding cardiac (dys)function. However, the protective mechanisms of HA in the cardiovascular system will require functions experiments.

## Methods

### Care and treatment of mice

AGAT^−/−^ mice were generated as previously described^14^. Mice used in this study were obtained from heterozygous breeding after backcrossing to a C57BL/6 J genetic background for at least six generations. All analysed animals were littermates. The mice (<5 per cage) were kept in standard cages under a 12 h:12 h light:dark cycle, constant temperature and humidity and received standard food and water ad libitum. Animal chow was essentially free of creatine^14^. Previously, no creatine and phosphocreatine could be detected in the heart of AGAT^−/−^ mice^15^. HA supplementation was achieved with osmotic mini pumps for 4 weeks, as previously described.^12^. Creatine supplementation was achieved by addition of 1% creatine to chow (Ssniff) as previously described^14^. All experimental procedures were approved by the respective local animal ethics committees (Behörde für Gesundheit und Verbraucherschutz Hamburg, approval no. 110/10) and investigations applied to the animal model were conformed to the guidelines for the care and use of laboratory animals published by the NIH (Publication No. 85–23, revised 1985).

### Tissue collection and preparation

Tissue collection and preparation were performed as previously described^14^. Briefly, mice were anesthetized with 2–3% isoflurane in 100% oxygen. After median thoracotomy the left ventricle of the heart was extracted, and shock frozen in liquid nitrogen for storage at −80 °C. Prior to use, frozen tissue was powdered with a steel mortar and pestle in liquid nitrogen.

### RNA isolation from murine tissue

RNA isolation was performed using QIAzol lysis reagent. Briefly, frozen tissue powder was minced in QIAzol and further disrupted using a pellet pestle. To extract RNA, chloroform was added, mixed, and centrifuged. The aqueous phase containing the RNA was collected and isopropanol was added. For precipitation, the RNA solution was centrifuged 15 min at 4 °C at high speed. After washing with 80% ethanol twice, the RNA pellet was dissolved in nuclease-free water. RNA concentration was determined by measuring absorbance at 260 nm using Nanodrop and stored at −80 °C until utilization.

### Gene expression analysis by microarray

Total RNA from left ventricular heart tissue was prepared in four groups of mice: WT (n = 7), AGAT^−/−^ (n = 7), AGAT^−/−^HA (n = 5) and AGAT^−/−^Cr (n = 4) mice. All groups of mice were analysed at the same time. RNA integrity was assessed on a 2100 Agilent Bioanalyzer (Agilent Technologies, Germany). The Affymetrix Mouse GeneChip ST 1.0 Array was used to assess the gene expression profile. Briefly, cRNA synthesis, labelling, fragmentation, array hybridization, washing and staining, and microarray scanning (Affymetrix GeneChip 3000 scanner) was performed according to manufacturer’s instruction of the Ambion WT Expression Kit and the Affymetrix GeneChip WT Terminal Labelling and Hybridization Kit with an input of 250 ng high quality RNA (RIN > 8).

### Reverse transcription and quantitative polymerase chain reaction (qPCR)

Reverse transcription and qPCR were performed as previously described^39^. One µg of total RNA was reverse transcribed using the High Capacity Kit (Life Technologies). For reverse transcription, samples were incubated for 2 hours at 37 °C followed by an inactivation step of 5 minutes at 85 °C. Finally, cDNA was diluted in water to a final concentration of 5 ng/µL. The relative quantification of mRNA levels was carried out on a 7900 TaqMan system (Applied Biosystems). To assess mRNA expression of target genes, real-time PCR was performed using 5 µL of the gene expression master mix (Thermo Fisher) and 0.5 µL of the gene expression assay (see Supplementary Table S4). Each gene expression assay includes forward and reverse primers as well as the FAM-labelled probe. As template 2 µL of cDNA was used in a final volume of 10 μL to detect. Each sample was analysed in duplicates and normalized to 18 S rRNA as endogenous control.

### Network analysis

Clusters (modules) of highly correlated genes were identified by weighted correlation network analysis (WGCNA)^40^. The so-called *eigengene* was computed for each module by calculating the first principal component based on all genes in the respective module. Associations between *eigengenes* of each module and genotype (WT and AGAT^−/−^) were calculated to identify modules highly de-regulated in AGAT^−/−^ compared to WT mice.

### WEB-based GEne SeT AnaLysis Toolkit (WebGestalt)

WebGestalt (www.webgestalt.org/) is a publicly available analysis toolkit for functional genomic, proteomic and large- scale genetic studies from which large number of gene lists are continuously generated^41^. Pathway analyses (WikiPathways) of differentially expressed gene sets were performed in order to identify an enrichment of genes in metabolic or disease-related pathways. The gene sets are adjusted for multiple testing based on the Benjamini-Hochberg method and a significance level of ≤0.05 was set for statistical significance.

### Gene expression omnibus (GEO)

To identify specific gene expression changes in MI, a systematic search in the GEO database^42^ was carried out. The keywords used for the GEO database search were: “myocardial infarction” AND “expression profiling by array” for “study type” AND “tissue” for “attribute name” AND “mus musculus” for “organism”. The GEO dataset GSE775^17^ was selected, which is a time series (1 hour, 4 hours, 24 hours, 48 hours, 1 week, and 8 weeks) intended to compare normal functioning left ventricles with infarcted left ventricles in mice. Expression profiling was performed based on the Affymetrix Murine Genome U74A Version 2 Array. MI has been mimicked by permanent ligation of the left coronary artery. Group comparison was performed using hearts subjected to MI (ilv) or the sham operation (lv and lv2). Raw data (CEL file) were downloaded and bioinformatics analysis were performed as described in the subsection “bioinformatics analysis”.

### Bioinformatics analysis

Differential gene expression analyses of murine transcriptomes were performed using the statistical language R and R/Bioconductor (www.bioconductor.org) packages *xps* and *limma*. Microarrays were pre-processed using the package *xps*. The *rma* function was used for background correction and normalization in order to reduce variation between arrays. The detection above background (DABG) was calculated for all genes and samples, and only genes with a DABG P value < 0.01 in at least two samples per group were kept for further analysis. The moderated t-test function *eBayes* from the *limma* package was used to calculate differential gene expression between groups. The False-Discovery-Rate (FDR) based Benjamini-Hochberg method was used to account for multiple tests, and the significance level for differentially expressed genes was set to a FDR ≤ 0.05.

### Statistical analysis

The mRNA levels were quantified according to the 2^−∆∆Ct^ method by Livak and Schmittgen^43^. For comparison of multiple groups, nonparametric Kruskal-Wallis-Test was used due to small sample sizes. Differences were considered statistically significant at a value of P ≤ 0.05. All calculations were performed using Graph Pad Prism 7.

## Supplementary information

Supplementary Information.

Supplementary Information2.

## Data Availability

Data, related documents such as study protocol and statistical analysis will be shared upon request from any qualified investigator for 3 years after the date of publication.
