# Supplementary material for: Analysis of L-arginine:glycine amidinotransferase-, creatine- and homoarginine-dependent gene regulation in the murine heart
Source: Sci Rep. 2020 Mar 16;10:4821. doi: 10.1038/s41598-020-61638-3 (PMC7076046; doi:10.1038/s41598-020-61638-3)
Supplement: Supplementary file 1 — Supplementary Information. [file 41598_2020_61638_MOESM1_ESM.pdf]

Analysis of L-arginine:glycine amidinotransferase-, creatine- and homoarginine-dependent gene regulation in the murine heart

Märit Jensen[1,3], Christian Müller[1,2], Chi-un Choe[2,3], Edzard Schwedhelm[2,4], Tanja Zeller[1,2*]

[1] University Heart and Vascular Centre Hamburg, Clinic for Cardiology, University Medical Centre Hamburg-Eppendorf, Hamburg, 20246, Germany

[2] German Centre for Cardiovascular Research (DZHK e.V.), partner site Hamburg/Kiel/Lübeck, 20246, Germany

[3] Department of Neurology, University Medical Centre Hamburg-Eppendorf, Hamburg, 20246, Germany

[4] Institute of Clinical Pharmacology and Toxicology, University Medical Centre Hamburg-Eppendorf, Hamburg, 20246, Germany

*Corresponding author:

Tanja Zeller

University Heart and Vascular Centre Hamburg, Clinic for Cardiology, University Medical Centre Hamburg-Eppendorf, Hamburg, 20246, Germany

t.zeller@uke.de

Phone: +49 (0) 40 7410 56575

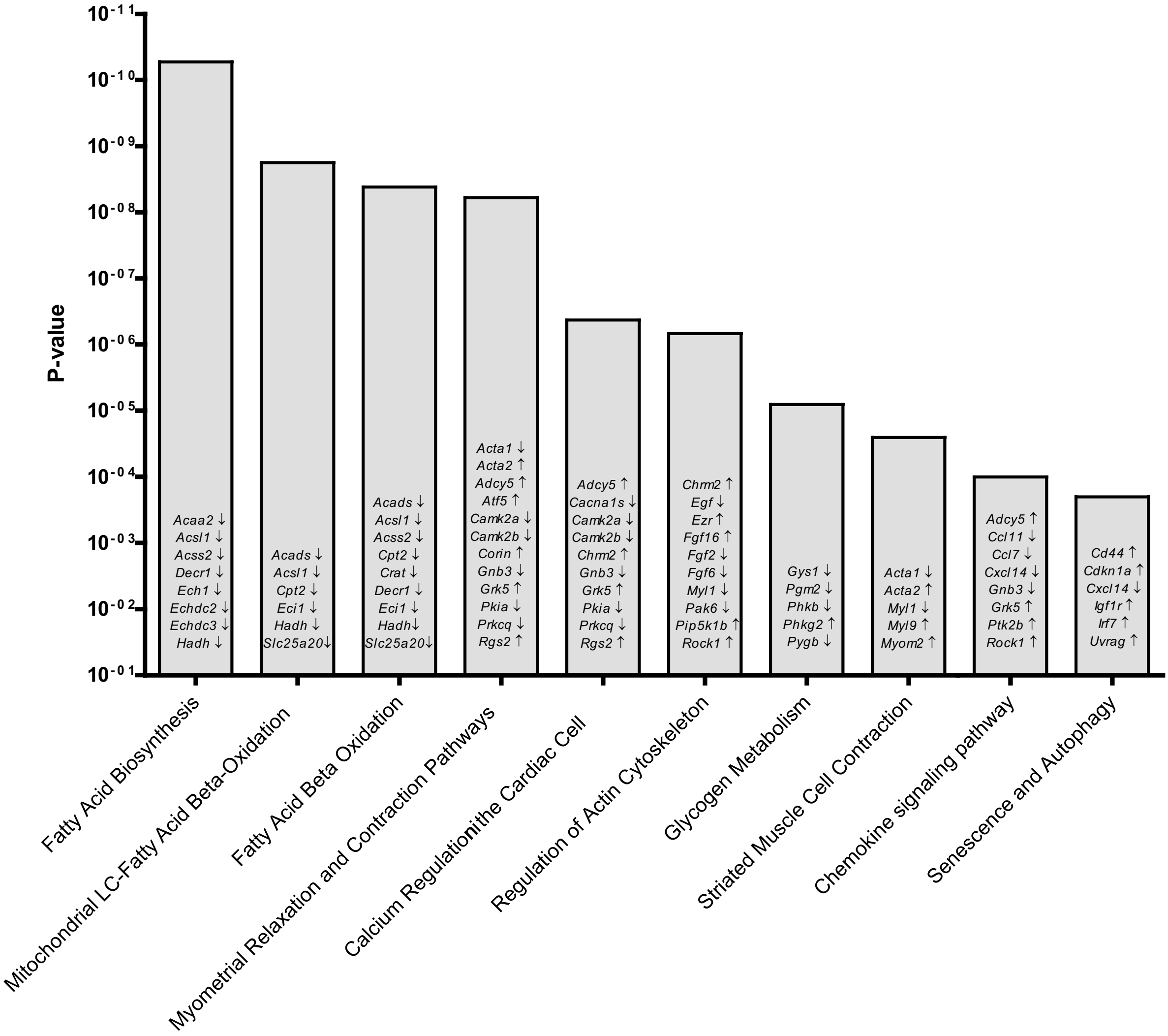

Supplementary Figure S1. Pathway analysis (WebGestalt) within the 485 significantly regulated genes between WT and AGAT$^{-/-}$ mice in heart tissue. The top ten pathways are shown, and the involved genes are listed in the bars. The direction of regulation is indicated by up and down arrows, respectively. P value ≤ 0.05.

Supplementary Table S2. Network analysis (Network 1) within differentially expressed genes between WT and AGAT$^{-/-}$ mice. The influence of creatine and HA is indicated by the comparison of WT and AGAT$^{-/-}$HA/Cr. False-Discovery-Rate ≤ 0.05. Abbreviations: FC, fold change; NS, not significant.

Gene	Gene name	WT vs AGAT$^{-/-}$		WT vs AGAT$^{-/-}$Cr		WT vs AGAT$^{-/-}$HA	
		**P Value**	**FC**	**P Value**	**FC**	**P Value**	**FC**
Scn4a	sodium channel, type IV, alpha	3.02×10^{-13}	-3.22	NS	-1.13	6.63×10^{-11}	-2.81
Scn4b	sodium channel, type IV, beta	1.47×10^{-12}	-3.74	NS	1.12	6.13×10^{-8}	-3.34

Tmod4	tropomodulin 4	8.93×10^{-11}	-1.76	NS	2.17	6.27×10^{-8}	-1.74
Tmem150c	transmembrane protein 150C	9.97×10^{-11}	-2.96	NS	-1.12	8.76×10^{-10}	-2.94
Fah	fumarylacetoacetate hydrolase	1.08×10^{-10}	-2.36	NS	-1.09	3.36×10^{-9}	-2.33
Lgi1	leucine-rich repeat LGI family, member 1	2.07×10^{-10}	-3.1	NS	1.06	9.99×10^{-10}	-4.27
Lad1	ladinin	2.63×10^{-10}	2.17	NS	1.01	2.23×10^{-9}	2.42
Stom	stomatin	1.12×10^{-9}	-1.41	NS	-1.05	7.04×10^{-9}	-1.46
Zfp106	zinc finger protein 106	1.31×10^{-9}	1.49	3.68×10^{-5}	1.33	2×10^{-7}	1.41
Egf	epidermal growth factor	6.62×10^{-9}	-1.55	NS	-1.15	2.86×10^{-7}	-1.46
Vwa8	von Willebrand factor A domain containing 8	1.2×10^{-8}	-1.35	2.65×10^{-2}	-1.1	6.77×10^{-6}	-1.27
Ano5	anoctamin 5	1.78×10^{-8}	-2.27	1.51×10^{-2}	-1.42	2.22×10^{-7}	-2.27
Ivd	isovaleryl coenzyme A dehydrogenase	2.54×10^{-8}	-1.41	NS	-1.06	1.11×10^{-7}	-1.53
Acsm5	acyl-CoA synthetase medium-chain family member 5	4.32×10^{-8}	-1.88	NS	-1.2	1.63×10^{-6}	-1.69
Ehd4	EH-domain containing 4	6.03×10^{-8}	1.36	NS	-1.04	3.15×10^{-6}	1.34
Myot	myotilin	7.62×10^{-8}	-2.36	NS	1.18	7.11×10^{-10}	-2.88
Sema4d	sema domain, immunoglobulin domain, transmembrane domain and short cytoplasmic domain, 4D	8.91×10^{-8}	-1.36	2.76×10^{-2}	-1.13	2.4×10^{-7}	-1.43
Abcc8	ATP-binding cassette, sub-family C (CFTR/MRP), member 8	1.03×10^{-7}	1.33	NS	1.03	8.64×10^{-7}	1.39
Igsf1	immunoglobulin superfamily, member 1	1.03×10^{-7}	-1.48	NS	-1.1	1.63×10^{-7}	-1.58
Cpt2	carnitine palmitoyltransferase 2	1.13×10^{-7}	-1.27	NS	1.02	4.52×10^{-6}	-1.28
Smim4	small integral membrane protein 4	1.5×10^{-7}	1.32	NS	1.02	1.26×10^{-5}	1.27
Pex11a	peroxisomal biogenesis factor 11 alpha	3.05×10^{-7}	-1.41	NS	-1.02	4.1×10^{-6}	-1.41
Acaa2	acetyl-Coenzyme A acyltransferase 2	5.5×10^{-7}	-1.22	NS	-1.04	1.2×10^{-4}	-1.18
Corin	corin	7.77×10^{-7}	1.36	NS	-1.07	4.64×10^{-6}	1.37
Mme	membrane metallo endopeptidase	7.88×10^{-7}	-1.72	3.86×10^{-2}	1.18	1.25×10^{-6}	-1.56
Ldhd	lactate dehydrogenase D	8.15×10^{-7}	-1.29	NS	-1.02	8.15×10^{-7}	-1.37
Prune2	prune homolog 2 (Drosophila)	8.62×10^{-7}	-1.56	NS	1.04	1.5×10^{-6}	-1.46
Slc25a20	solute carrier family 25, member 20	9.42×10^{-7}	-1.31	NS	-1.03	1.53×10^{-6}	-1.38
Spta1	spectrin alpha, erythrocytic 1	1.79×10^{-6}	1.43	NS	1.01	1.31×10^{-5}	1.42
Gys1	glycogen synthase 1, muscle	2.21×10^{-6}	-1.29	NS	1.01	6.01×10^{-7}	-1.41
Cenpf	centromere protein F	4.76×10^{-6}	1.9	NS	-1.15	3.11×10^{-5}	1.98
Bckdha	branched chain ketoacid dehydrogenase E1, alpha polypeptide	6.49×10^{-6}	-1.23	NS	1.02	5.34×10^{-4}	-1.18
Ncam1	neural cell adhesion molecule 1	7.68×10^{-6}	1.42	NS	-1.13	9.55×10^{-5}	1.4
Adig	adipogenin	1.15×10^{-5}	-1.32	NS	1.02	1.39×10^{-5}	-1.39
Lmf1	lipase maturation factor 1	3.63×10^{-5}	-1.17	NS	1.01	3.51×10^{-4}	-1.17
Adam9	a disintegrin and metallopeptidase domain 9 (meltrin gamma)	9.79×10^{-5}	-1.16	NS	-1.02	2.86×10^{-4}	-1.17
Slc25a35	solute carrier family 25, member 35	1.05×10^{-4}	-1.15	NS	1.12	1.06×10^{-4}	-1.25

Supplementary Table S3. Network analysis (Network 2) within differentially expressed genes between WT and AGAT$^{-/-}$ mice. The influence of creatine and HA is indicated by the comparison of WT and AGAT$^{-/-}$HA/Cr. False-Discovery-Rate ≤ 0.05. Abbreviations: FC, fold change; NS, not significant.

Gene	Gene name	WT vs. AGAT$^{-/-}$		WT vs AGAT$^{-/-}$Cr		WT vs AGAT$^{-/-}$HA	
		P Value	FC	P Value	FC	P Value	FC
Rpp25	ribonuclease P/MRP 25 subunit	8.71×10^{-8}	1.69	NS	1	2×10^{-5}	1.57
Fbp2	fructose bisphosphatase 2	1.06×10^{-7}	-1.57	1.64×10^{-2}	-1.12	4.66×10^{-7}	-1.46
Naa35	N(alpha)-acetyltransferase 35, NatC auxiliary subunit	1.32×10^{-7}	1.33	NS	1.04	2.64×10^{-4}	1.2
Clcn1	chloride channel 1	2.22×10^{-7}	-1.57	NS	1.01	3.03×10^{-4}	-1.37
2210407C18Rik	RIKEN cDNA 2210407C18 gene	2.31×10^{-7}	-2.39	1.41×10^{-2}	1.44	9.46×10^{-5}	-2.25
Fgl2	fibrinogen-like protein 2	3.1×10^{-7}	1.44	2.69×10^{-3}	-1.2	3.65×10^{-2}	1.15
Ucp2	uncoupling protein 2	3.49×10^{-7}	1.85	NS	-1.13	8.67×10^{-6}	1.53
Ctgf	connective tissue growth factor	5.21×10^{-7}	1.78	3.1×10^{-3}	-1.33	1.36×10^{-7}	1.83
Stk39	serine/threonine kinase 39	7.93×10^{-7}	1.4	NS	-1.01	3.91×10^{-4}	1.29
Trim7	tripartite motif-containing 7	1.51×10^{-6}	-1.37	NS	1.02	1.16×10^{-3}	-1.28
Gpam	glycerol-3-phosphate acyltransferase, mitochondrial	1.92×10^{-6}	-1.6	NS	-1.09	1×10^{-6}	-1.5
Hk1	hexokinase 1	3.64×10^{-6}	1.28	NS	1.03	2.73×10^{-4}	1.21
Lrrc51	leucine rich repeat containing 51	9.01×10^{-6}	1.23	NS	1.04	1.32×10^{-3}	1.19
Nppa	natriuretic peptide type A	1.17×10^{-5}	1.93	NS	1.02	1.16×10^{-2}	1.55
Klhl13	kelch-like 13	1.26×10^{-5}	1.31	1.03×10^{-2}	-1.15	2.75×10^{-2}	1.17
Hcn2	hyperpolarization-activated, cyclic nucleotide-gated K+ 2	1.48×10^{-5}	1.33	9.67×10^{-3}	-1.17	2.57×10^{-3}	1.2
Pla2g5	phospholipase A2, group V	1.97×10^{-5}	1.26	4.64×10^{-2}	-1.12	2.27×10^{-2}	1.13
Tmem100	transmembrane protein 100	2×10^{-5}	1.56	NS	1	1.12×10^{-4}	1.47
Perp	PERP, TP53 apoptosis effector	3.08×10^{-5}	1.2	NS	1	8.06×10^{-4}	1.18
Ezr	ezrin	3.69×10^{-5}	1.25	NS	-1.09	1.74×10^{-5}	1.26
Dsc2	desmocollin 2	4.33×10^{-5}	1.35	NS	-1.07	NS	1.10
Rock1	Rho-associated coiled-coil containing protein kinase 1	5.68×10^{-5}	1.23	NS	1.03	8.07×10^{-4}	1.22
Camk2n1	calcium/calmodulin-dependent protein kinase II inhibitor 1	5.92×10^{-5}	1.27	NS	1.06	6.36×10^{-4}	1.24
Polq	polymerase (DNA directed), theta	7.66×10^{-5}	-1.31	NS	1.01	1.24×10^{-3}	-1.27
Ppm1e	protein phosphatase 1E	8.5×10^{-5}	1.34	NS	1.02	1.89×10^{-3}	1.27
Slc4a4	solute carrier family 4, member 4	9.44×10^{-5}	1.19	3.14×10^{-2}	-1.09	4.62×10^{-3}	1.15
Slc25a42	solute carrier family 25, member 42	1.33×10^{-4}	-1.29	NS	-1.07	4.75×10^{-3}	-1.15
F3	coagulation factor III	1.8×10^{-4}	1.24	NS	-1.03	1.01×10^{-3}	1.21

Ehbp1	EH domain binding protein 1	2×10^{-4}	1.16	NS	1	4.98×10^{-3}	1.13
Zdhhc2	zinc finger, DHHC domain containing 2	2.05×10^{-4}	1.39	NS	-1.02	9.46×10^{-4}	-1.17
Ldb3	LIM domain binding 3	2.65×10^{-4}	-1.15	NS	-1.02	1.48×10^{-2}	-1.13
Capn6	calpain 6	2.81×10^{-4}	1.19	NS	1.02	NS	1.08
Hcn4	hyperpolarization-activated, cyclic nucleotide-gated K+ 4	2.93×10^{-4}	-1.42	NS	-1.07	8.8×10^{-3}	-1.28
Ghitm	growth hormone inducible transmembrane protein	3.23×10^{-4}	1.14	NS	1.02	3.07×10^{-4}	1.18
Gcnt1	glucosaminyl (N-acetyl) transferase 1, core 2	3.42×10^{-4}	1.21	NS	1.11	NS	1.07
Uvrag	UV radiation resistance associated gene	3.45×10^{-4}	1.14	NS	-1.03	NS	1.05
2310007L24Rik	RIKEN cDNA 2310007L24 gene	4.28×10^{-4}	-1.2	NS	-1.01	NS	-1.07
Trpm7	transient receptor potential cation channel, subfamily M, member 7	4.66×10^{-4}	1.24	NS	1.01	4.64×10^{-4}	1.25
Nt5c2	5'-nucleotidase, cytosolic II	5.03×10^{-4}	1.2	NS	-1.1	NS	1.09
Pgm5	phosphoglucomutase 5	5.07×10^{-4}	1.16	NS	1	3.45×10^{-2}	1.11
Slc38a1	solute carrier family 38, member 1	5.54×10^{-4}	1.23	NS	-1.01	2.06×10^{-1}	1.09
Nomo1	nodal modulator 1	6.03×10^{-4}	1.13	NS	-1.02	NS	1.07
Nt5dc2	5'-nucleotidase domain containing 2	6.1×10^{-4}	1.15	NS	-1.14	NS	1.1
Peli1	pellino 1	6.1×10^{-4}	1.22	NS	1.09	NS	1.12
Nebl	nebulette	6.4×10^{-4}	1.14	NS	1	7.7×10^{-3}	1.13
Ubxn6	UBX domain protein 6	6.49×10^{-4}	1.13	NS	-1.01	1.8×10^{-3}	1.16
R3hcc1	R3H domain and coiled-coil containing 1	6.56×10^{-4}	1.16	NS	-1.04	NS	1.08
Hipk1	homeodomain interacting protein kinase 1	1.04×10^{-3}	1.16	NS	-1.01	2.65×10^{-3}	1.17
Sh3bp4	SH3-domain binding protein 4	1.06×10^{-3}	1.2	NS	1.05	NS	1.12

Supplementary Table S4. Gene expression assays used for qPCR.

Gene	Assay ID
18S rRNA	Hs99999901_s1
Ctgf	Mm00515790_g1
Dsc2	Mm00516355_m1
Ehd4	Mm00505588_m1
Fbp2	Mm00484280_m1
Fgl2	Mm00433327_m1
Gatm (Agat)	Mm01268678_m1
Hcn2	Mm00468538_m1
Hcn4	Mm01176086_m1
Nppa	Mm01255748_g1
Rpp25	Mm00547632_s1
Scn4a	Mm00500103_m1
Scn4b	Mm01175562_m1
Sema4d	Mm00443147_m1
Ucp2	Mm00627599_m1
Uvrag	Mm00724370_m1
